# Age as a Prognostic Factor in Anaplastic Thyroid Cancer

**DOI:** 10.1155/2014/240513

**Published:** 2014-06-15

**Authors:** Vladan Zivaljevic, Katarina Tausanovic, Ivan Paunovic, Aleksandar Diklic, Nevena Kalezic, Goran Zoric, Vera Sabljak, Berislav Vekic, Rastko Zivic, Jelena Marinkovic, Sandra Sipetic

**Affiliations:** ^1^Center for Endocrine Surgery, Clinical Center of Serbia, 11000 Belgrade, Serbia; ^2^School of Medicine, Belgrade University, 11000 Belgrade, Serbia; ^3^Surgical Clinic, KBC Dedinje, 11000 Belgrade, Serbia

## Abstract

*Background.* Anaplastic thyroid cancer (ATC) is one of the tumors with the shortest survival in human medicine. * Aim.* The aim was to determine the importance of age in survival of patients with ATC. * Material and Methods*. We analyzed the data on 150 patients diagnosed with ATC in the period from 1995 to 2006. The Kaplan-Meier method and log-rank test were used to determine overall survival. Prognostic factors were identified by univariate and multivariate Cox regression analysis. * Results.* The youngest patient was 35 years old and the oldest was 89 years old. According to univariate regression analysis, age was significantly associated with longer survival in patients with ATC. In multivariate regression analysis, patients age, presence of longstanding goiter, whether surgical treatment is carried out or not, type of surgery, tumor multicentricity, presence of distant metastases, histologically proven preexistent papillary carcinoma, radioiodine therapy, and postoperative radiotherapy were included. According to multivariate analysis, besides surgery (*P* = 0.000, OR = 0.43, 95% CI = 0.29–0.63), only patients age (*P* = 0.023, OR = 0.68, 95% CI = 0.49–0.95) was independent prognostic factor of favorable survival in patients with ATC. * Conclusion*. Age is a factor that was independently associated with survival time in ATC. Anaplastic thyroid cancer has the best prognosis in patients younger than 50 years.

## 1. Introduction

Anaplastic thyroid cancer (ATC) is one of the most aggressive tumors in human medicine. This aggressiveness is reflected in the local infiltrative growth and early metastatic spread. Therefore, the prognosis of ATC patients is one of the worst among the malignant tumors in general.

Despite multimodal treatment, which includes surgery, radiotherapy, and chemotherapy, survival time is discouraging.

Fortunately, ATC is a rare tumor. Its annual incidence is about 2 per million residents and its share in the structure of patients with thyroid cancer is decreasing and now stands at 1-2% [1, 2]. ATC occurs most frequently in the elderly but has been described in all age groups [3, 4].

As one of the most aggressive tumors, ATC draws a lot of attention, but it has not been sufficiently researched. Due to its low incidence, there are not many institutions that have single series with more than 100 patients. Analysis of prognostic factors is possible only in this larger series because most patients unfortunately die in the first few weeks or months after diagnosis, so one might get wrong impression that there is no significant survival in patients with ATC. In larger series, significant survival was recorded, which enables analysis of prognostic factors associated with longer survival in patients with ATC.

The aim of this study was to determine the role and importance of age in survival of patients with ATC.

## 2. Material and Methods

We analyzed retrospectively the data on 150 patients with ATC (96 women and 54 men) diagnosed in the period from 1995 to 2006. In 85 operated-on patients, the diagnosis was obtained on the basis of definitive histopathological finding, and in 65 nonoperated-on patients diagnosis was based on cytological findings after fine needle aspiration. Patients with histologically proven preexisting papillary thyroid carcinoma (49) were also included in the study.

Reviewing medical records, we collected data on demographic characteristics of patients (age, sex, place of residence, and life on endemic goiter area), duration of disease before diagnosis, whether the patients knew of goiter before and if so for how long back, and whether the operation was done or just fine needle aspiration biopsy. In the operated-on patients, the following has been analyzed: the type of surgery that was done, then the size of the tumor, its multicentricity, the involvement of one or both lobes of the thyroid gland, the presence of regional lymph node metastases, or distant metastases proven.

Special attention was focused on the histopathological findings in the operated-on patients and the presence of preexisting well-differentiated thyroid cancer, as well as the degree of lymphocytic infiltration of the tumor.

Data on whether in the operated-on patients radiotherapy (preoperative or postoperative), chemotherapy, or radioactive iodine therapy was implemented has been gathered.

Some other diseases in patients with anaplastic thyroid cancer which could have an impact on survival were taken into consideration, other malignant tumors, diabetes, and hypertension. Basic demographic and clinical characteristics of ATC patients are shown in Table 1.

Data on whether patients were still alive or when they died were gathered through interviews of patients or their families.

The Kaplan-Meier method and log-rank test were used to determine the overall survival. Potential prognostic factors were identified by univariate and multivariate Cox regression analysis. SPSS (version 17.0) was used for statistical analysis.

## 3. Results

Distribution of patients with anaplastic thyroid carcinoma is presented in Table 2.

The largest number of patients with anaplastic thyroid carcinoma was in the seventh decade of life (more than half of the patients) and then in the sixth and eighth decade of life (almost 20%). Under 40 years of age, anaplastic carcinoma was detected in just two affected (1.3%) and in younger than fifty in 11 patients (7.3%). The youngest patient was 35 years old and the oldest was 89 years old. The average age of patients was 64 years (standard error ± 8.5 years).

**Figure 1:**
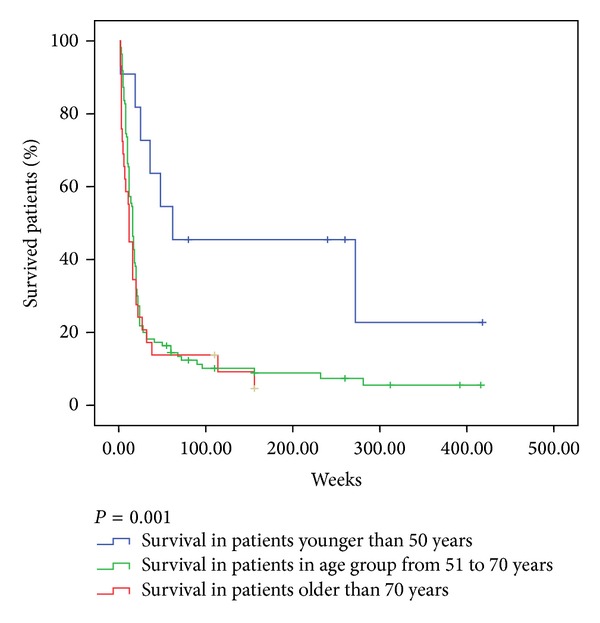
Kaplan-Meier survival curve in patients with anaplastic thyroid cancer in relation to age.

Table 3 and Figure 1 show the survival rates of patients with anaplastic carcinoma of the thyroid gland in relation to the age. The patients were divided into three age groups: the first consisted of patients younger than 51 years, the second consisted of patients aged 51 to 70 years, and the third consisted of patients older than 70 years. The three age groups <50 ys, 51–70 ys, and >70 were arbitrarily chosen.

The longest survival in all periods was found in the youngest age group and the lowest survival in the oldest group of patients in which no one had a survival of more than 3 years. In the youngest age group, one-year survival was observed in more than half of the patients, whereas in both the older age groups one-year survival was 3-4 times less. During the first month from diagnosis of anaplastic thyroid carcinoma, almost 30% of patients older than 70 years died and less than 10% in the two younger age groups. Five-year survival rates were three times more often in the youngest age group than in the middle one. In the oldest group, there were no patients with the five-year survival rate. The middle and the oldest age group were compared for other characteristics described in Material and Methods section, but the observed differences did not affect the survival according to multivariate analysis.

Mean survival and median survival in patients with anaplastic carcinoma of the thyroid gland in relation to age are presented in Table 4. In the youngest age group the median survival was 62 weeks and in the middle age group 16 weeks, which was the median for all patients with anaplastic thyroid cancer overall, while the shortest median of 12 weeks was found in the oldest age group of patients. This difference was statistically highly significant according to the log-rank test (*P* = 0.008).

According to univariate Cox regression analysis, among the factors that influence the survival of patients with anaplastic thyroid cancer, patients age was significantly statistically associated with longer survival (*P* = 0.000, OR = 0.96, 95% CI = 0.94–0.99). All the factors associated with longer survival in patients with ATC at the level of statistical significance (*P* < 0.1) according to univariate model were then included in the model of multivariate analysis: patients age, presence of longstanding goiter before the appearance of ATC, surgery, type of surgery, tumor multicentricity, presence of distant metastases at the time of diagnosis, histologically proven preexistent papillary carcinoma, radioiodine therapy, and postoperative radiotherapy. According to multivariate analysis, after eliminating the confound effect, besides surgery (*P* = 0.000, OR = 0.43, 95% CI = 0.29–0.63), only patients age (*P* = 0.023, OR = 0.68, 95% CI = 0.49–0.95) was independent prognostic factor of favorable survival in patients with ATC.

## 4. Discussion

The average age of patients with ATC in our study was 64 years, and more than half of the patients were in their seventh decade of life. Only two affected were younger than 40 years of age, and the youngest patient was a 35-year-old woman. Our oldest patient with anaplastic thyroid cancer was an 89-year-old man. According to the literature, the largest number of patients with anaplastic carcinoma of the thyroid gland is in the seventh and eighth decade of life [5]. The average age of patients ranges from 65 to 72 years, and there is no significant difference between men and women in relation to the age at which anaplastic thyroid cancer usually occurs [6, 7]. More than 90% of patients in all the major series are older than 50 years [5, 8]. The oldest person with anaplastic thyroid cancer was 104 years old [6]. Anaplastic thyroid cancer is rare under 40 years of age [9]. In childhood, its appearance is quite rare and has been described only in few cases in the world. Unfortunately, in the childhood, it has the same fatal outcome, as well as in adult patients [10–12]. Age of patients with anaplastic thyroid cancer at the time of diagnosis, according to our results, is an independent factor that was significantly associated with longer survival (*P* < 0.05, RR = 0.65, 95% CI = 0.49 to 0.95). In other words, survival is significantly longer in younger than in older patients. However, it should be noted that in the youngest age category of patients with ATC (younger or at the age of 50) in whom the best survival was found, there is only 11 patients. Almost half of the patients died during the first year (5/11); a five-year survival was observed only in two patients.

According to the literature, data on the impact of age on survival in patients with ATC are controversial, although longer survival can be expected in younger patients, and it is known that anaplastic thyroid cancer usually occurs in older patients [13]. Besic et al. [14] point out that in patients with anaplastic thyroid cancer, who are older than 70 years, the risk of shorter survival is 1.5 times higher than in patients younger than 70 years, but the age is not an independent predictor according to the multivariate regression analysis. Yau et al. [6] found that survival was significantly longer in patients younger than 65 years, as a reason for cited comorbidity that is present in the elderly. Giuffrida and Gharib [15] and Kebebew et al. [16] found longer survival in patients with anaplastic thyroid cancer who are younger than 60 years. Gilliland et al. [17] found that one-year survival in patients with anaplastic thyroid cancer declines in older patients. Li and colleagues [18] by analyzing 12 patients with ATC under the age of 55 found that the prognosis is better than in older patients but only if the remaining of histologically proven preexistent papillary carcinoma exists. Sugitani et al. [19] in the largest of all studies of prognostic factors in ATC, which included 677 ATC patients from 38 institutions, in multivariate regression analysis among other factors independently associated with a worse prognosis state also the age over 70 years. Unlike the most, according to the results by Haigh et al. [20], years of age had no influence on survival in patients with anaplastic thyroid carcinoma.

According to our study, besides patient age, only surgery was independent prognostic factor of favorable survival in patients with ATC. Among the nonoperated-on patients, no one survived longer than one year, and among the operated-on ones the mean survival rate was seven times higher. However, the independent predictor significantly associated with survival is the type of operation performed, that is, how radical the operation was. As many as one-half of the patients that underwent radical surgery lived longer than one year and one-fourth lived longer than five years. Yau et al. [6] found a significant link between surgical treatment and the length of survival. In their series, 68% of the patients underwent surgical treatment, and half of them had complete removal of the tumor. The results of Haigh et al. [20] prove that radical surgical treatment is an independent survival predictor, and it was possible to carry it out in 31%. According to literature, radical surgical treatment is an independent survival predictor [6, 20]. The best results are obtained by multimodal treatment [5, 8, 13]. However, some of the authors did not find that multimodal treatment had an impact on the survival, where the only positive prognostic factor was the complete resection of the tumor [7, 21]. Their study, like ours, showed that none of the nonoperated-on patients reached the point of one-year survival. Multiannual survival in patients with complete resection of the tumour occurs irrespective of the extent of operation on the thyroid, that is, irrespective of the fact whether a total thyroidectomy or only hemithyroidectomy was performed, the only condition being that the tumour was totally removed [11]. According to Venkatesh et al. [13], surgery is vital but not sufficient enough for longer survival, as one-year survival is noted in operated-on patients but almost never in nonoperated-on ones, and radical operation without multimodal treatment does not significantly prolong the survival. The mean survival of patients, who undergo multimodal treatment, is 11 months [5].

## 5. Conclusion

Age is one of the most important factors in the survival of patients with ATC. The best prognosis has patients younger than 50 years, but the smallest number of patients is in this age category. The limitation of our study is relatively small number of patients although it is one of the single institution series with the greatest number of patients in the world. Nevertheless, the results of this study should be verified as many cases as possible by conducting multicenter study out of different centers in the world.

## Figures and Tables

**Table 1:** Demographic and clinical characteristics of patients with anaplastic thyroid cancer.

Variables	Number	Percent (%)
Gender		
Male	54	36.0
Female	96	64.0
Residence in endemic goiter area	11	11.3
Goiter	53	35.5
Duration of goiter		
0	97	64.7
<10	18	12.0
10–20	18	12.0
>20	17	11.3
Surgery		
Yes	85	56.7
No	65	43.3
Type of surgery		
Open biopsy	12	14.1
Tracheotomy	2	2.4
Tumor reduction	27	31.8
Lobectomy	4	4.7
Thyroidectomy and dissection	40	47.1
Multicentricity of tumor	5	6.0
Presence in one or both thyroid lobes		
One	58	68.2
Both	27	31.8
Tumor size		
T1–T3	3	3.6
T4	82	96.4
Metastases in lymph nodes	54	63.5
Distant metastases	28	32.9
Preexisting well-differentiated tumor (papillary)	49	57.6
Lymphocyte infiltration	7	8.2
Preoperative radiotherapy	2	2.4
Postoperative radiotherapy	67	78.7
Chemotherapy	2	78.7
Radioactive iodine therapy	8	9.4
Other malignant tumors	6	7.1
Diabetes	13	15.3
Arterial hypertension	26	30.6

**Table 2:** Distribution of patients with anaplastic thyroid cancer according to age.

Age groups (years)	Number	Percent (%)
≤40	2	1.3
41–50	9	6.0
51–60	29	19.3
61–70	81	54.0
>70	29	19.3

Total	150	100.0

**Table 3:** Survival rates in patients with anaplastic thyroid cancer according to age.

Follow-up period (weeks)	The survival rate for patients under the age of 50 years (%)	The survival rate in patients with 51–70 years (%)	The survival rate in patients older than 70 years (%)
4 weeks	90.0	91.8	72.4
16 weeks	81.8	46.4	34.5
52 weeks	54.5	16.4	13.8
260 weeks	22.7	7.4	0

**Table 4:** Mean and median survival in patients with anaplastic thyroid cancer (in weeks) in relation to age.

Age (years)	Mean survival (weeks)	Standard error	95% confidence interval	Median survival (weeks)	Standard error	95% confidence interval
<50	174.3	52.7	71–277.5	62.0	86.6	0–231.8
51–70	50.2	10.1	30.5–70.1	16.0	1.1	13.8–18.2
>70	30.0	8.8	12.8–47.3	12.0	0.9	10.2–13.7

Log-rank *P* = 0.008.
